# A novel crumbs homolog 1 mutation in a family with retinitis pigmentosa, nanophthalmos, and optic disc drusen

**Published:** 2012-10-04

**Authors:** Codrut C. Paun, Benjamin J. Pijl, Anna M. Siemiatkowska, Rob W.J. Collin, Frans P.M. Cremers, Carel B. Hoyng, Anneke I. den Hollander

**Affiliations:** 1 Department of Human Genetics, Radboud University Nijmegen Medical Centre, Nijmegen, The Netherlands; 2 Department of Ophthalmology, Radboud University Nijmegen Medical Centre, Nijmegen, The Netherlands; 3 Nijmegen Centre for Molecular Life Sciences, Radboud University Nijmegen Medical Centre, Nijmegen, the Netherlands

## Abstract

**Purpose:**

The purpose of this study is to identify the genetic defect in a Turkish family with autosomal recessive retinitis pigmentosa, nanophthalmos, and optic disc drusen.

**Methods:**

Ophthalmological examinations consisted of measuring the best-corrected visual acuity and the refractive error, electroretinography, optical coherence tomography, B-mode ultrasonography, and fundus photography. The involvement of the membrane frizzled-related protein (*MFRP*) gene in this family was studied with direct DNA sequencing of the coding exons of *MFRP* and with linkage analysis with microsatellite markers. After *MFRP* was excluded, genome-wide homozygosity mapping was performed with 250 K single nucleotide polymorphism (SNP) microarrays. Mutation analysis of the crumbs homolog 1 (*CRB1*) gene was performed with direct sequencing.

**Results:**

Ophthalmological evaluation of both affected individuals in the family revealed a decreased axial length (18–19 mm), retinal dystrophy, macular edema, and hyperopia of >+8.0 diopters. Sequencing of *MFRP* did not reveal any pathogenic changes, and microsatellite marker analysis showed that the chromosomal region did not segregate within the disease in this family. Genome-wide homozygosity mapping using single nucleotide polymorphism microarrays revealed a 28-Mb homozygous region encompassing the *CRB1* gene, and direct sequencing disclosed a novel homozygous missense mutation (p.Gly833Asp) in *CRB1*.

**Conclusions:**

Previous studies associated mutations in the *MFRP* gene with the syndrome nanophthalmos-retinitis pigmentosa-foveoschisis-optic disc drusen. In this study, we demonstrated that a similar disease complex can be caused by mutations in the *CRB1* gene.

## Introduction

Nanophthalmos-retinitis pigmentosa-foveoschisis-optic disc drusen disease complex has been described as a distinct recessive entity [1,2]. The disease can be described as characteristically having a short axial eye length (13.0–18.5 mm), high hyperopia (+8.00 to +25.00 diopters), retinal pigment epithelium atrophy, formation of optic disc drusen, and foveoschisis [3]. Mutations in the membrane frizzled related protein (*MFRP*) gene were described as responsible for causing the disease complex [1–3]. The *MFRP* gene is located on chromosome 11q13 and encodes a membrane receptor protein specifically expressed in the retinal pigment epithelium and ciliary epithelium of the eye [4]. MFRP is thought to play a role in eye development, as mutations in the gene that codes for this protein have been associated with nanophthalmos, retinitis pigmentosa (RP), and other degenerative disorders [5,6].

In this study, we describe the clinical and genetic features of a consanguineous Turkish family with two affected siblings with RP, nanophthalmos, and optic disc drusen. We excluded the involvement of the *MFRP* gene in the family, and report a novel mutation in *CRB1*, a gene previously associated with autosomal recessive RP and Leber congenital amaurosis [7].

## Methods

### Clinical analyses

Ophthalmological examinations of the affected siblings included measuring the best corrected visual acuity (BCVA) and refractive error, electroretinography (ERG) according to the International Society for Clinical Electrophysiology of Vision (ISCEV) protocol [8], B-mode ultrasonography, fundus photography, and spectral domain optical coherence tomography (SD-OCT). Because of the early-onset and severity of the disease, the unaffected parents who had no complaints were not subjected to the ophthalmological examinations.

### Genetic analyses

We obtained blood samples and pedigree information after receiving informed consent from all individuals. Approval was obtained from the institutional review board. Genomic DNA was isolated from lymphocytes with automated DNA extraction (Hamilton ML Star, Hamiloton Bonaduz AG, Bonaduz, Switserland).

Primers were designed using Primer3 online software. All of the coding exons and the exon/intron boundaries of *MFRP* were amplified with polymerase chain reaction (PCR) using the primers contained in Table 1.

**Table 1:** Primers used for amplification and sequence analysis of the *MFRP, RBP3 and RP1* gene.

**Gene**	**Exon**	**Sequence (5′-3′)**	
*MFRP*	1	F: CCCCCACACAGAGACAGAGT	R: CTGGTGCTGGGTCTTAGGAG
	2 and 3	F: CTCCTAAGACCCAGCACCAG	R: TCATGGAGTTTCATTCCAAAGC
	4 and 5	F: ACCCAGCTCCTCTGAACGC	R: GATAGTGGTTCAGGACACGG
	6 and 7	F: CTGACCCTGCTCTTGGAGC	R: CTTGAACCCAGATCAGACGC
	8 and 9	F: ATGGAGGCACAGATCCTAGC	R: ACAGTGAGGATGGAGTTATCC
	10 and 11	F: GTCAGCCAGGGCTGGTGC	R: GCACCCAGCCTGCTCAGG
	12 and 13	F: AGAGCCAGTGAGCAGTCCC	R: GACCGGCAAAAGAGGACG
	13	F: AGCTGACCTGGAAGCTTGTG	R: GCAGAGAGATGAGGGTGGAG
*RBP3*	1, fragment 1	F: CTTGCACACAGTCCAGGGAG	R: AGATCCAGCACTAAGGCGG
	1, fragment 2	F: TGGAGGGTAATGTGGGCTAC	R: GTCCCCACACAGGGCAG
	1, fragment 3	F: GCTGAGGATAGGCGAGTCTG	R: CGGAGGCGTCAGCAAAAC
	1, fragment 4	F: CTGAGGACGAGGCTATCCG	R: TTGTCGATGAAGGTGAGGAC
	1, fragment 5	F: CTTCCTTATGCAGTCGCTGG	R: TCAAAACGCAGGTAGCCC
	1, fragment 6	F: CGAGCTGGTGGTAGAGGAAG	R: TGCATATAAGGGGCTGCTG
	1, fragment 7	F: CTTTGCACACACCATGCAG	R: CAATGGGTCAACTCACTCCC
	2	F: CTGGGCTCTAAAACTGGCTG	R: GCCCATAGCTTTGACTGTCC
	3	F: GCACACAGGGCCTCACTG	R: CTGTCTTTCCCTGGTTTCCC
	4	F: GAGAAGACAGGTGCTCCAGG	R: GGTGTGTGTCCCAGAGGTTC
*RP1*	1	F: CCATGTATTCGCTATGGTGC	R: TGTCCAGGTCTACAGGCTGC
	2	F: GGCAGGCACAGCATCAC	R: CACCATTCATATCCCACACG
	3	F: TTCAAGCCTAGGAGGTTGTTG	R: ATTGAAGCATGGATTTTGCC
	4, fragment 1	F: GATATTTCTAACTTCTCTGCCTTCC	R: CCCTGGATGATATCTGTGTCC
	4, fragment 2	F: ATCAAGAGGGCAGTTTGGC	R: TTGAAGTTCTTGATACCAGTTTTG
	4, fragment 3	F: TCACATAATAATGGTTTGCCATC	R: TTTCTATGGAAATTCTTGGAAATC
	4, fragment 4	F: TCCCCTTAAAGGAGGGATAC	R: AATTGAATGATGAGCAATAGCC
	4, fragment 5	F: GAATGGCAAAGAAGAGTTTAGTTTC	R: ACTGAAGCTTGCAATTGGTG
	4, fragment 6	F: GCTTATTTGGTTCCCCTGC	R: AGAGCAACCTCCATCCAAAG
	4, fragment 7	F: ACTTGAAAGCTGCTGTTGCC	R: GCTTAAATTACTGACATTTTGATGTG
	4, fragment 8	F: CAATGTCTGCAATACCATTGAC	R: TCCTTCATTGGTCTCCTTTTC
	4, fragment 9	F: TTAATCCAAGAAGAGGTAGAGGC	R: CCTGGAATTCCTGCAACATAG
	4, fragment 10	F: TGGAATTTCAGTGTTCCAGG	R: TGATGACTACCCTTCTCCTCTG
	4, fragment 11	F: CATGGTAGTGACTCAGAACCTTTTC	R: CCTTCTTCCTCTAACCCCAAG
	4, fragment 12	F: GATAATGCCATTGGTGATATATTTG	R: CGTATTCGTCACATGTGCTTC

PCR products were purified with gel extraction (QIAquick Gel Extraction Kit; Qiagen, Venlo, the Netherlands) or with 96-well filter plates (MultiScreen HTS-PCR; Millipore, Bedford, MA). Bidirectional dideoxy sequencing was performed using the forward and reverse primers (BigDye Terminator, ver. Three on a 3730 or 3100 DNA Analyzer; Applied Biosystems, Inc., [ABI], Foster City, CA). Sequencing results were analyzed with Vector NTI (Invitrogen Life Technologies Europe BV, Bleiswijk, the Netherlands) software. The microsatellite markers used for linkage analysis are presented in Table 2.

**Table 2:** Microsatellite markers used for haplotype analysis at the *MFRP* locus.

**Chromosome**	**Position (hg18)**	**Name**	**D number**
chr11	118,140,606–118,140,889	AFMA222XC5	D11S4104
chr11	118,884,802–118,884,982	AFMB342ZE9	D11S4171
chr11	120,333,420–120,333,756	AFM220YB6	D11S925

DNA samples of both affected individuals were genotyped with 250 K single nucleotide polymorphism (SNP) microarrays (GeneChip Mapping 250 K Nsp Array; Affymetrix, Santa Clara, CA). Array experiments were performed according to protocols provided by the manufacturer. Arrays were scanned, and genotypes were called as described [9]. The 250 K SNP data were analyzed with the software package CNAG [10], and chromosomal segments were accepted as homozygous if the loss of heterozygosity (LOH) score was ≥10. The LOH score measures the likelihood of a stretch of SNPs being homozygous based on the population SNP allele frequencies. An LOH score of ≥15 corresponds to regions of (on average) 4 Mb and larger [11]. Homozygous regions shared by both individuals were analyzed for the presence of known RP genes. Retinol-binding protein 3 (*RBP3*) and retinitis pigmentosa 1 (*RP1*) were screened for mutations as described above for *MFRP*. *CRB1* amplification and sequencing were performed as described previously [7]. Turkish controls were screened for the novel mutation in *CRB1*, with restriction enzyme digestion with BccI.

## Results

### Clinical findings

**Figure 1:**
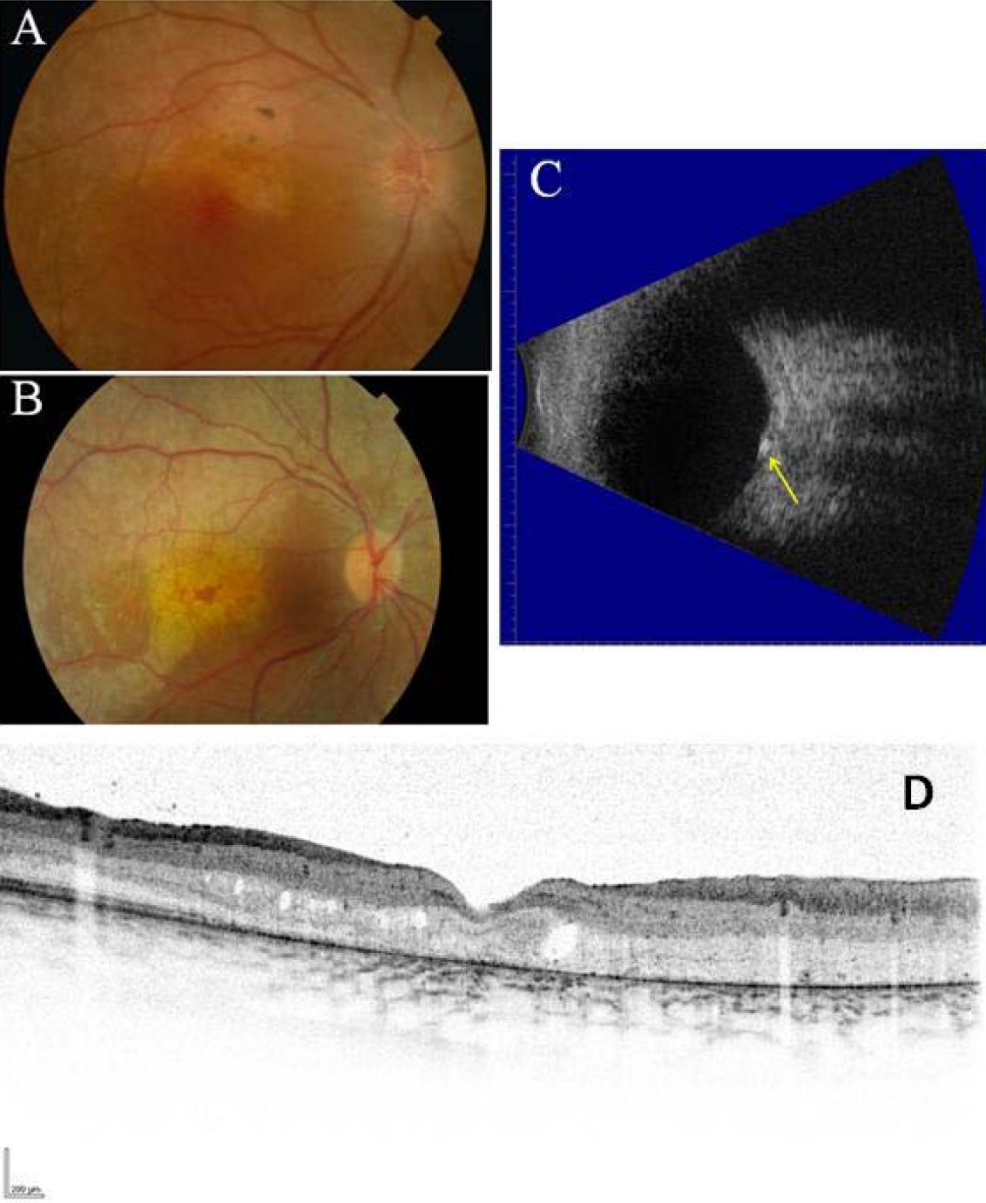
Ophthalmological images of two siblings affected by retinitis pigmentosa, nanophthalmus and optic disc drusen. Fundus photography of the right eye of patient IV:2 **A**: and of patient IV:3 **B**: showed atrophy of the retina outside the fovea and spots of hyperpigmentation. B-mode ultrasound of the left eye of patient IV:2 **C**: revealed optic disc drusen (indicated with the yellow arrow). Optical coherence tomography scan of the macula of patient IV:2 showed intraretinal edema and atrophy of the outer retinal layers **D**.

Table 3 summarizes the ophthalmologic features of both affected individuals. Both patients demonstrated bilateral decreased axial length, retinal dystrophy, and macular edema (Figure 1). Patient IV:2 had optic disc drusen on funduscopy and confirmed by B-mode ultrasound, whereas patient IV:3 did not have optic disc drusen. The ERG for patient IV:3 showed an extinguished rod response and a subnormal cone photopic response. In patient IV:2, the rod and cone responses were extinguished on the ERG.

**Table 3:** Clinical characteristics of affected members of a family with retinitis pigmentosa, nanophthalmos and optic disc drusen.

**Patient**	**Age (years)**	**BCVA (Snellen)**	**Axial length (mm)**	**Refractive error (D)**	**Posterior Segment Findings**	**Ultrasound**	**OCT findings**
IV:2	17	0.20 OD	19.47 OD	+8.25 OD	Atrophy of the retina outside of the fovea, spots of hyperpigmentation	Optic disc drusen	Intraretinal macular edema
		0.16 OS	19.46 OD	+7.75 OS			
IV:3	7	0.10 OD	18.86 OD	+9.5 OD	Atrophy of the retina outside of the fovea, spots of hyperpigmentation	No optic disc drusen	Intraretinal macular edema
		0.10 OS	18.89 OS	+9.25 OS			

BCVA=best-corrected visual acuity; OD=right eye; OS=left eye; D=diopters; OCT=optical coherence tomography

### Genetic findings

**Figure 2:**
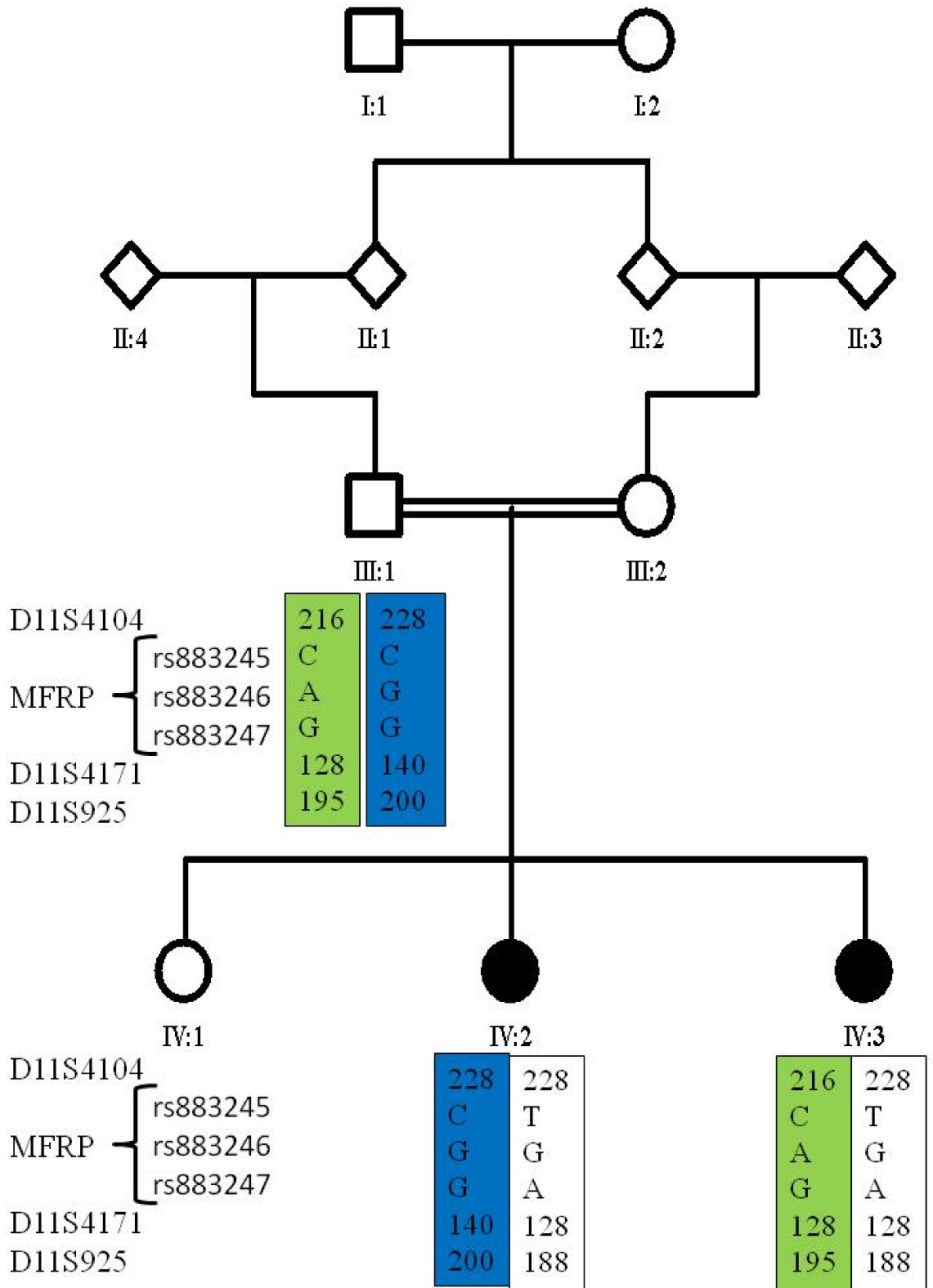
Exclusion of the membrane frizzled-related protein gene in a family with retinitis pigmentosa, nanophthalmos, and optic disc drusen with haplotype analysis. Haplotypes were constructed using microsatellite markers and single nucleotide polymorphisms detected in exon 1 of the membrane frizzled-related protein gene.

Direct sequencing of the *MFRP* gene in the proband (patient IV:2) did not reveal a disease-causing mutation. The only detected variations were known SNPs in exons 1, 4, and 5 (Table 4). Haplotypes were constructed based on microsatellite markers and SNPs at the *MFRP* locus (Figure 2). Both affected individuals inherited different chromosomal haplotypes from their father at this locus, excluding involvement of this locus in this family.

**Table 4:** Sequence variants identified by sequence analysis of the *MFRP* and *RP1* gene.

**Gene**	**Exon**	**cDNA**	**Protein**	**SNP number**
*MFRP*	1	c.-88C>T	-	rs883245
	1	c.-65G>A	-	rs883246
	1	c.-31G>A	-	rs883247
	4	c.406G>A	p.Val136Met	rs3814762
	5	c.540T>C	p.His180=	rs2510143
	5	c.492C>T	p.Tyr164=	rs36015759
*RP1*	4	c.5175A>G	p.Gln1725=	rs441800
	4	c.2615G>A	p.Arg872His	rs444772
	4	c.5071T>C	p.Ser1691Pro	rs414352

Genome-wide homozygosity mapping using SNP microarrays revealed several homozygous regions in both patients. Thirteen regions were shared in both, with the largest region spanning 28.8 Mb on chromosome 1. Analysis of the shared homozygous regions for the known RP genes revealed that the *CRB1* gene resided in the largest homozygous segment, and two other known RP genes (*RP1* and *RBP3*) were present in smaller homozygous regions (Table 5). Sequence analysis of the *RP1* and *RBP3* genes in the proband (patient IV:2) revealed only nonpathogenic SNPs in exon 4 of *RP1* (Table 4).

**Table 5:** Homozygous regions shared by patients IV:2 and IV:3 identified by genome-wide SNP microarray analysis.

**Chromosome**	**Size (Mb)**	**Start position (hg18)**	**End position (hg18)**	**Number of homozygous SNPs**	**RP Gene**
1	28.8	167,580,132	196,437,697	2788	*CRB1*
12	20.2	12,966,664	33,172,827	2440	
8	20.0	20,080,993	40,179,228	1901	
10	18.3	29,819,314	48,158,305	1334	*RBP3*
4	15.1	147,053,197	162,160,056	1516	
8	13.7	42,830,763	56,575,870	855	*RP1*
4	12.1	84,301,279	96,462,585	1123	
11	8.1	47,323,947	55,496,802	307	
19	6.3	49,894,480	56,209,610	207	
12	5.2	5,127,842	10,400,609	450	

**Figure 3:**
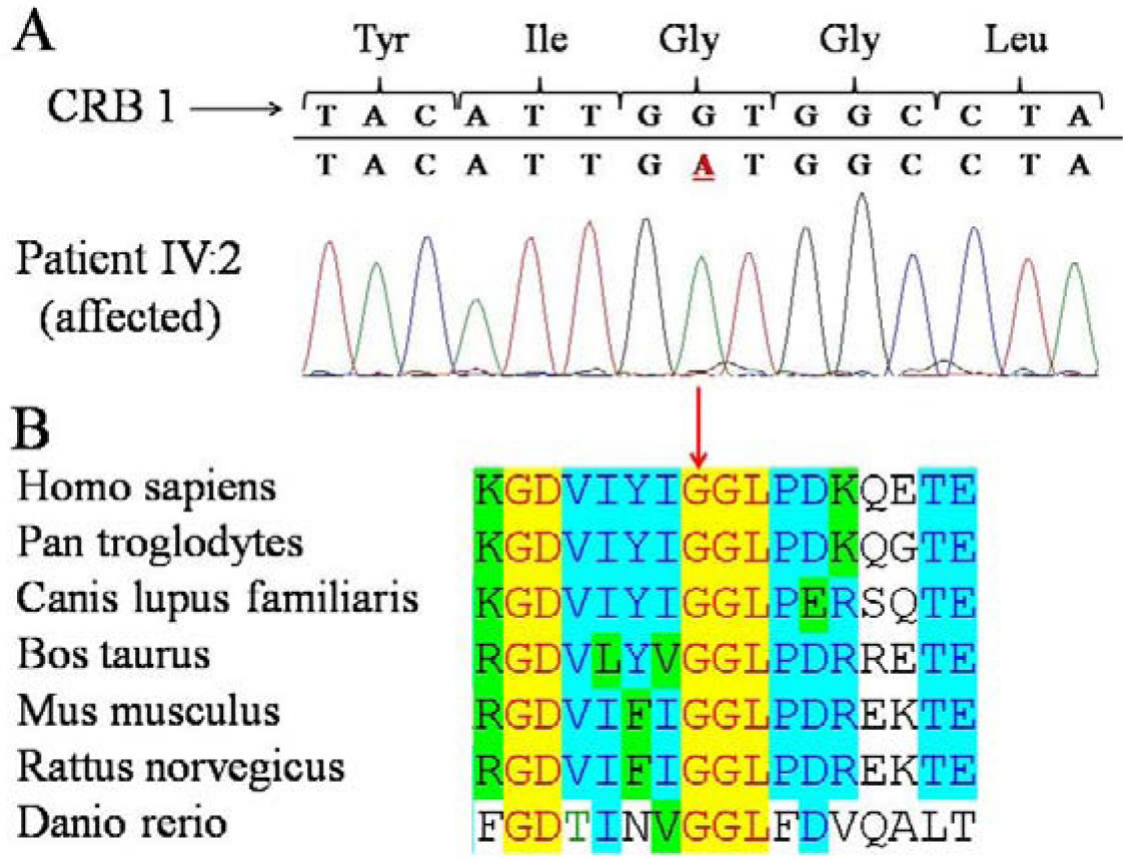
Identification of a novel crumbs homolog 1 mutation in a family with retinitis pigmentosa, nanophthalmos, and optic disc drusen. **A**: Crumbs homolog 1 sequence analysis demonstrated a homozygous mutation (c.2498G>A) in exon 7. **B**: At the protein level, the mutation (p.Gly833Asp) alters a highly conserved amino acid residue.

Sequence analysis of *CRB1* revealed a novel homozygous missense mutation in exon 7 (c.2498G>A; p.Gly833Asp), which affects a highly conserved amino acid residue (Figure 3). The mutation was found homozygously in both affected siblings, and heterozygously in the unaffected father. Restriction enzyme digestion did not reveal the mutation in 100 Turkish controls. Bioinformatic analyses confirmed pathogenicity of the mutation (Grantham score: 94, Sorting Intolerant From Tolerant [SIFT]: deleterious, Polymorphism Phenotyping v2 [PolyPHen-2]: probably damaging with a score of 1.000, PhyloP: 5.3).

## Discussion

Several research groups have described mutations in the *MFRP* gene, leading to an autosomal recessive disease characterized by nanophthalmos, RP, foveoschisis, and optic disc drusen [1–3]. In this study, we demonstrate that a similar disease complex can be caused by a novel missense mutation in the *CRB1* gene. This is in agreement with a recent study that identified a homozygous *CRB1* mutation in a Mexican family with similar features [12].

In both individuals of the family described in this study, we observed a decreased axial length consistent with nanophthalmos, resulting in high hyperopia. High hyperopia is commonly seen in patients with *CRB1* mutations [13,14]. Optic disc drusen were observed in patient IV:2, but not in patient IV:3, which may be due to her young age (7 years) at examination [15]. On OCT, we noted a similar cystic appearance as observed in patients with *MFRP* mutations in previous studies [1–3], but in our opinion, this does not resemble former publications of classical foveoschisis [16]. More likely, the patients developed macular edema secondary to RP, resulting in a split appearance of the macula on OCT.

The involvement of *MFRP* was excluded in this family, and homozygosity mapping revealed a novel missense mutation in the *CRB1* gene. The mutation resides in the second laminin A G-like domain, where the mutation affects a residue in a highly conserved region and localizes near several other missense mutations previously identified in *CRB1* [17]. Our results demonstrate that mutations in not only *MFRP* but also *CRB1* are associated with small eye size. The combination of features observed in this family closely resembles the nanophthalmos- retinitis pigmentosa-foveoschisis-optic disc drusen disease complex previously associated with *MFRP* mutations.
